# Supplementary material for: Splice-Junction-Based Mapping of Alternative Isoforms in the Human Proteome
Source: Cell Rep. Author manuscript; Available in PMC 2020 Jan 15. (PMC6961840; doi:10.1016/j.celrep.2019.11.026)
Supplement: 2 [file NIHMS1546469-supplement-2.zip › DF1/PXD010154/Liver/Liver_28_AGXT_VTTSPGPPKALLKPLSIPNQLLLGPGPSNLPPR.pdf]

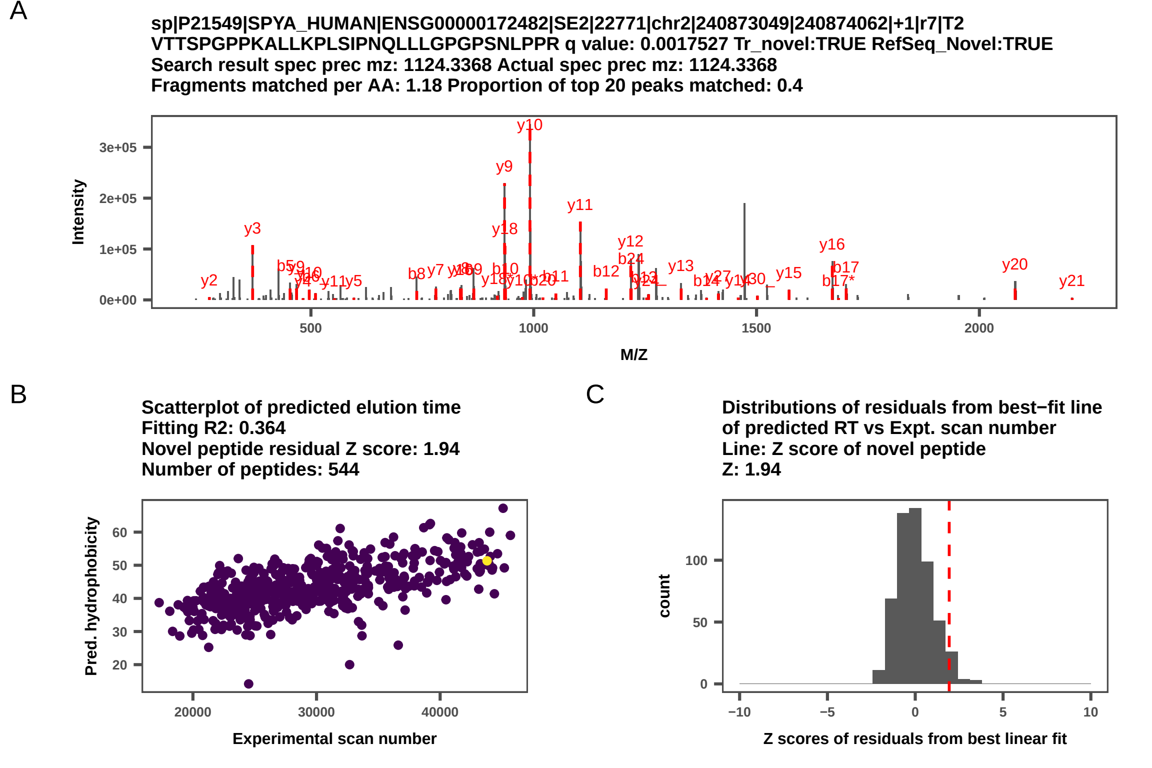
A
sp|P21549|SPYA_HUMAN|ENSG00000172482|SE2|22771|chr2|240873049|240874062|+1|r7|T2
VTTSPGPPKALLKPLSIPNQLLLGPGPSNLPPR q value: 0.0017527 Tr_novel:TRUE RefSeq_Novel:TRUE
Search result spec prec mz: 1124.3368 Actual spec prec mz: 1124.3368
Fragments matched per AA: 1.18 Proportion of top 20 peaks matched: 0.4
Intensity
M/Z
B
Scatterplot of predicted elution time
Fitting R2: 0.364
Novel peptide residual Z score: 1.94
Number of peptides: 544
Pred. hydrophobicity
Experimental scan number
C
Distributions of residuals from best−fit line
of predicted RT vs Expt. scan number
Line: Z score of novel peptide
Z: 1.94
count
Z scores of residuals from best linear fit
